# Prevalence and aetiology of moderate and severe thrombocytopenia in a tertiary and quaternary centre in KwaZulu-Natal

**DOI:** 10.4102/ajlm.v9i1.799

**Published:** 2020-08-24

**Authors:** Ayanda G.P. Jali, Bongani B. Nkambule

**Affiliations:** 1 Department of Haematology, Health King Edward VIII Hospital, University of Kwa-Zulu Natal, Durban, South Africa; 2 Department of Haematology, National Health Laboratory service, Inkosi Albert Luthuli Academic Hospital, Durban, South Africa; 3 Department of Human Physiology, School of Laboratory Medicine and Medical Sciences, University of KwaZulu-Natal, Durban, South Africa

**Keywords:** thrombocytopenia, prevalence, aetiology, South Africa, haematology

## Abstract

**Background:**

Thrombocytopenia is a common haematological disorder, characterised by platelet counts below 150 × 10^9^/L. The aetiology of thrombocytopenia is multifactorial; notably, in a misdiagnosis this condition may be due to pre-analytical laboratory artefacts. Knowledge about the common aetiology of thrombocytopenia will assist clinicians in decision-making and interpretation of laboratory tests and this may lead to prompt, adequate patient management and cost-saving measures.

**Objective:**

This study determined the prevalence and aetiology of moderate and severe thrombocytopenia in a tertiary or quaternary laboratory in Durban, KwaZulu-Natal, South Africa.

**Methods:**

We conducted a retrospective study at the Inkosi Albert Luthuli Central Hospital haematology laboratory between October 2015 and April 2016. A total of 2076 full blood count results with a platelet count of less than 100 × 10^9^/L were retrieved from the Inkosi Albert Luthuli Academic Hospital database. Laboratory data were extracted and matched with clinical data and used to identify the potential aetiology of thrombocytopenia.

**Results:**

The prevalence of thrombocytopenia was 14.9% within the selected study period. The haematology or oncology wards and clinic accounted for 55.2% of thrombocytopenia cases, whereas the adult and paediatric intensive care units accounted for 29.3%. Notably, 15.5% of thrombocytopenia cases were reported in non-haematology wards and clinics. The most common cause of thrombocytopenia was chemotherapy which accounted for 38.5% of all causes.

**Conclusion:**

In our tertiary and quaternary setting, thrombocytopenia in adults was most common in patients admitted to haematology and oncology wards. Moreover, chemotherapy-induced thrombocytopenia accounted for more than a third of all these cases.

## Introduction

Thrombocytopenia is a common clinical condition that is associated with multiple systemic diseases. Thrombocytopenia is characterised by platelet counts below 150 × 10^9^/L. However, only platelet counts of less than 100 × 10^9^/L are considered clinically significant.^1^ Moderate thrombocytopenia is defined as a platelet count range of 50–100 × 10^9^/L, while severe thrombocytopenia is classified by a platelet count of less than 50 × 10^9^/L.^2^ The aetiology of thrombocytopenia varies and may be caused by mild to life-threatening clinical conditions. However misdiagnosis of thrombocytopenia also occurs, and could be the result of artefactual laboratory errors.^3,4^ A full blood count and peripheral blood smear are mandatory as an initial test in patients with thrombocytopenia.^1,5^ Although there have been advances in the automation of haematology analysers, the peripheral blood smear still remains an important diagnostic tool.^1^ It provides an informed interpretation of the full blood count results, as there are morphological abnormalities that an automated analyser cannot detect.^3,6^ In particular, it helps to distinguish between thrombocytopenia due to laboratory errors and true thrombocytopenia.^3^ Thrombocytopenia can be caused by decreased thrombopoiesis in the bone marrow and increased sequestration of platelets by the spleen as a result of malignancy, aplastic anaemia, myelodysplastic syndrome and opportunistic infections.^1,7^ Another aetiology of thrombocytopenia is increased peripheral destruction of platelets that may occur following disseminated intravascular coagulation, thrombotic microangiopathy and increased platelet sequestration due to hypersplenism.^4,8^

For prompt and adequate management of patients with thrombocytopenia, a comprehensive review of the patients’ medical records should be performed by the treating clinician, and appropriate physical examination and investigations need to be performed.^1^ Previous studies have shown an increased prevalence of thrombocytopenia in hospitalised patients in developing and developed countries.^4,9,10^ In patients with haematological malignancies receiving chemotherapy, thrombocytopenia as a consequence of drug toxicity levels has been reported.^12^ Understanding the aetiology of thrombocytopenia in hospitalised patients is pivotal, as thrombocytopenia may lead to complications in patients with a variety of conditions.^9,10,11^ In a previous study conducted in Johannesburg, South Africa in 2012, the authors reported on chemotherapy and sepsis as the common causes of thrombocytopenia, irrespective of HIV status.^4^ However, the prevalence and aetiology of thrombocytopenia in a tertiary or quaternary hospital setting remains unknown as data describing this remain scarce. The aim of this study was to determine the prevalence and clinical diagnosis associated with thrombocytopenia in patients presenting at Inkosi Albert Luthuli Central Hospital in KwaZulu-Natal between October 2015 and April 2016.

## Methods

### Ethical considerations

The study received ethical approval from the University of KwaZulu-Natal Biomedical Research and Ethics Committee (Approval number: BE 297/16).

### Sample selection and data extraction

This retrospective study was conducted at the Department of Haematology at Inkosi Albert Luthuli Central Hospital (IALCH), Durban, KwaZulu-Natal, South Africa. The department offers diagnostic haematology services to the entire province of KwaZulu-Natal. The IALCH is an 846-bed referral hospital that provides tertiary level services to the entire KwaZulu-Natal province and parts of the Eastern Cape.

### Data collection

Patients’ diagnostic test results and clinical notes were retrieved from the IALCH TRAK laboratory data management system, version 6.10.56 (Intersystems Corporation, Cambridge, Massachusetts, United States). The extracted laboratory data items included the peripheral blood film report, diagnosis, platelet count, ward number and hospitalisation status. Clinical data items were retrieved from Meditech version 615 (Meditech, Westwood, Massachusetts, United States). The extracted patient information included age, sex, type of ward, hospitalisation status and the cause of thrombocytopenia in these patients.

**FIGURE 1:**
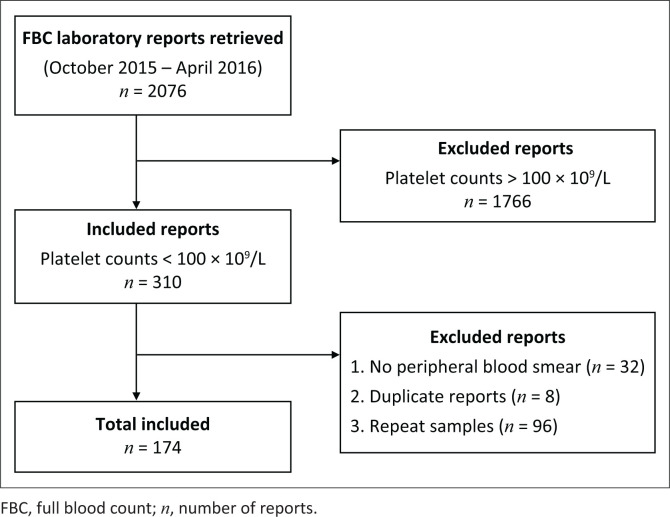
Study design. This study included full blood count reports retrieved from the Department of Haematology for patients presenting at the Inkosi Albert Luthuli Central Hospital, Durban, KwaZulu-Natal, South Africa (October 2015 – April 2016).

A total of 2076 full blood count results were extracted from the TRAK database. These results were from samples received at IALCH haematology laboratory on non-consecutive weekdays from October 2015 to April 2016. This was in an effort to minimise the inclusion of repeat samples in our analysis. All samples were analysed using the same Sysmex XE5000 analyser (Sysmex Corporation, Chuo-ku, Kobe, Japan). The study included patient reports that fulfilled the study inclusion criteria of a platelet count of < 100 × 10^9^/L and had a corresponding peripheral blood smear report (Figure 1).

### Sample size determination

In order to determine the prevalence of thrombocytopenia, we computed a minimum required sample size (*n*) of 304 based on the thrombocytopenia prevalence of 20% and an appropriate precision (*d*) of 0.05 and a 95% confidence interval width of 15.5% – 24.5%.^13^ Furthermore, a sub-sample size of 170 was required to estimate the aetiology of thrombocytopenia with a 5% probability of error and assuming that 50% of cases are due to acquired factors.

### Statistical analysis

The Kolmogorov-Smirnov and Lilliefors tests for normality were used to assess data distribution. Parametric data such as platelet count and cluster of differentiation 4 T-cell counts were reported as mean and standard deviations. Platelet counts were ordered into two categories (i.e. < 50 and 51–100). The Fisher’s exact test was used for comparisons between ordinal data (age, platelet count, diagnosis and race). The prevalence of thrombocytopenia was reported as a proportion of patients with the disease. Furthermore, associations between patient characteristics (age, sex and hospital ward) and aetiology of thrombocytopenia were assessed. A *p*-value of < 0.05 was regarded as statistically significant. All data analyses were performed using the Stata version 13.1 statistical software (StataCorp LP, College Station, Texas, United States).

## Results

A total of 2076 full blood count reports were retrieved from the IALCH database. Among these, 174 reports that met the inclusion criteria were included in the study (Figure 1). The extracted participant data included age, gender, peripheral blood film report, diagnosis, platelet count, ward number and hospitalisation status.

### Patient characteristics

The study comprised 174 thrombocytopenia patients with a mean age of 24.4 ± 2.9 years. This consisted of patients receiving both inpatient and outpatient care (Table 1). The HIV status of the thrombocytopenia patients was reported in 146 (83.9%) of the included cases, while it was not determined in 28 (16.1%) (Table 1). Thirty-one (21.2%) of the thrombocytopenia patients were HIV-positive with a mean cluster of differentiation 4 T-cell count of 261.3 ± 199.3 cells/*µ*L.

**TABLE 1:** Characteristics of patients presenting at the Department of Haematology Inkosi Albert Luthuli Central Hospital, Durban, KwaZulu-Natal, South Africa (October 2015 – April 2016).

Variable	*n*	%
**Age**		
0–12	65	37.4
13–24	38	21.8
25–36	26	14.9
37–48	17	9.8
49–60	17	9.8
> 60	11	6.3
**Sex**		
Male	85	48.9
Female	89	51.1
**Platelet count** (mean, SD)	49.9	30.5
**HIV status**		
Positive	31	17.8
Negative	115	66.1
Not determined†	28	16.1
**CD4 count** (cells/*µ*L mean, SD)‡	261.3	199.3
**Hospitalisation status**		
Inpatient	149	85.6
Outpatient	25	14.4

*n* = 174.

CD4, cluster of differentiation 4.

† , No laboratory results available.

‡ , CD4 counts of all HIV-positive patients.

### Prevalence and aetiology of moderate to severe thrombocytopenia

The overall prevalence of thrombocytopenia was 14.9%. The paediatric population (0–12 years) had the highest prevalence of thrombocytopenia (37.4%) compared with those aged 13–24 years (21.8%), 25–36 years (14.9%), 37–48 years (9.8%), 49–60 years (9.8%) and > 60 years (6.3%) (Table 1). In the overall cohort, the prevalence of thrombocytopenia was higher in female patients (51.1%) compared to male patients (48.9%), *p* < 0.001. Thrombocytopenia was most common in inpatients (85.6%) compared to outpatients (14.4%), *p* < 0.001. The adult and paediatric haematology and oncology wards and clinic had the highest prevalence of thrombocytopenia (55%), whereas the adult and paediatric intensive care units (29.3%) and the non-haematology wards and clinic (15.5%) had a lower prevalence, *p* < 0.001. Overall, severe thrombocytopenia (platelet count ≤ 50 × 10^9^/L) was twofold higher (64.9%) than moderate thrombocytopenia (35.1%), *p* = 0.004 (Table 2).

**TABLE 2:** Aetiology of thrombocytopenia in patients presenting at the Department of Haematology Inkosi Albert Luthuli Central Hospital, Durban, KwaZulu-Natal, South Africa (October 2015 – April 2016).

Parameter	Malignancies		Sepsis		Chemo		Pregnancy		ITP		Aplastic		Other		*p*-value
	*n*	%	*n*	%	*n*	%	*n*	%	*n*	%	*n*	%	*n*	%	
**Sex**															
Male	8	4.6	19	10.9	42	24.1	-	-	1	0.6	7	4.0	4.0	2.3	**< 0.001**
Female	12	6.9	29	16.7	25	14.4	9	5.2	7	4.0	1	0.6	14	8.0	
**Age group**															
0–12 years	6	3.4	35	20.1	14	8.0	0	-	0	-	3	1.7	7	4.0	ND
13–24 years	6	3.4	1	0.6	18	10.3	5	2.9	5	2.9	2	1.1	5	2.9	
25–36 years	4	2.3	5	2.9	8	4.6	4	2.3	2	1.1	2	1.1	3	1.7	
37–48 years	1	0.6	3	1.7	11	6.3	0	-	0	-	0	-	1	0.6	
49–60	1	0.6	3	1.7	10	5.7	0	-	1	0.6	1	0.6	1	0.6	
> 60	2	1.1	1	0.6	6	3.4	0	-	0	-	0	-	1	0.6	
**Platelet count**															
≤ 50	15	8.6	35	20.1	41	23.6	4	2.3	3	1.7	8	4.6	11	6.3	0.075
51–100	5	2.9	13	7.5	26	14.9	5	2.9	5	2.9	0	-	7	4.0	
**Patient**															
Inpatient	17	9.8	48	27.6	57	32.8	9	5.2	7	4.0	0	-	15	8.6	**< 0.001**
Outpatient	3	1.7	0	-	10	5.7	0	-	1	0.6	8	4.6	3	1.7	
Medical	20	11.5	44	25.3	67	38.5	5	2.9	8	4.6	8	4.6	15	8.6	**< 0.001**
Surgical	0	-	4	2.3	0	-	4	2.3	0	-	0	-	3	1.7	

**Total**	20	11.5	48	27.6	67	38.5	9	5.2	8	4.6	8	4.6	18	10.3	

ND, not determined due to low subgroup numbers.

Statistically significant results (*p* < 0.05) are shown in bold.

Chemo, chemotherapy; ITP, immune thrombocytopenic purpura; Aplastic, Aplastic anaemia.

Chemotherapy-induced thrombocytopenia (38.5%) and sepsis (27.6%) accounted for the majority of thrombocytopenia cases (Table 2). Malignancies accounted for 11.5%, immune thrombocytopenic purpura 4.6% and aplastic anaemia 4.6%. Aetiologies with a prevalence less than 4% were grouped into one category and these comprised trauma as well as pre-eclampsia, storage disorders, autoimmune disorders, hereditary thrombocytopenia and human leukocyte antigen antibodies to platelets, which accounted for between 0.6 and 1.7%.

## Discussion

In this study, we report a thrombocytopenia prevalence of 14.9%, which is higher than the 8.6% prevalence reported in a similar study conducted at an academic state hospital in Johannesburg, South Africa.^4^ This higher prevalence may be due to the fact that the current study was conducted at a tertiary and quaternary hospital, to which only specific cases are referred and this could result in an overestimation of the prevalence of thrombocytopenia. In addition, other studies have reported on seasonal and genetic variations in platelet counts.^14^ Taken together these factors may account for the differences in the prevalence of thrombocytopenia. In fact, seasonal variations in platelet counts have been reported with lower platelet counts observed in spring and summer, while slightly elevated platelet counts have been observed in autumn and winter.^14,15^ Notably our study period fell within a low thrombocytopenia season (summer and autumn); this may suggest that the reported platelet counts in patients with severe thrombocytopenia were not influenced by seasonal variations but may have led to an underestimation of thrombocytopenia in our setting. Notably, only 17.8% of the patients included in our study were HIV-positive compared to 36% reported in the previous retrospective study by Vaughan et al. reporting on patient full blood count reports authorised in 2012, at the Chris Hani Baragwanath Academic Hospital, South Africa.^4^ We further report on a higher frequency of thrombocytopenia in hospitalised patients compared to outpatients, which is similar to that previously described.^4^ This may be due to the differences in diagnosis and disease severity between admitted patients and outpatients. Moreover, in our study thrombocytopenia was particularly common among patients admitted to haematology and oncology wards, intensive care units, neonatal intensive care units and medical wards, while a minority of thrombocytopenia cases (1.7%) were from non-haematology clinics. These findings are consistent with those previously reported. Interestingly, in our study thrombocytopenia was common particularly among children below the age of 12 years and the majority of the cases were due to sepsis.

More than a third of cases referred to the haematology department with thrombocytopenia were classified as chemotherapy-induced. The mechanism of thrombocytopenia in chemotherapy-induced thrombocytopenia involves reduced platelet production, an increased rate of platelet destruction and enhanced platelet clearance by immune mechanisms.^7^ Bone marrow infiltration by malignancy can cause suppression of megakaryopoiesis, resulting in thrombocytopenia.^1,7^ In our study, malignancies accounted for more thrombocytopenia cases when compared to bone marrow failure. Contrary to our findings, a study conducted in Johannesburg, South Africa in 2012 showed that bone marrow failure accounted for 9.6% of cases while malignancies accounted for 7.4% of thrombocytopenia cases.^4^

Thrombocytopenia due to sepsis accounted for 27.6% of the cases, a majority of which were in the intensive care unit. The majority of cases presenting with sepsis had severe thrombocytopenia; these findings were similar to those previously described.^16^ Studies have shown that the prevalence of thrombocytopenia in sepsis ranges from 33.8% to 60%.^5,6,17^ Immune thrombocytopenic purpura, an isolated thrombocytopenia with no associated clinical conditions,^18^ was seen in 4.6% of the participants and most participants were between 13 and 24 years; the majority were in the obstetrics and labour ward (87.5%). In adults, immune thrombocytopenic purpura is a chronic disease resulting from an autoimmune disorder mediated by platelet antibodies, increased platelet destruction and impaired platelet production.^18,19^

Contrary to literature, immune thrombocytopenic purpura was not common among the paediatric group (12.5%).^19^ Neonatal thrombocytopenia commonly occurs as a result of increased platelet destruction or sequestration resulting from infections, respiratory distress syndrome or infants whose mothers had pre-eclampsia.^20^ Thrombocytopenia can also be the result of decreased platelet production in congenital abnormalities of the newborn such as thrombocytopenia absent radii.^20^ Thrombocytopenia can also be seen in children who are well and commonly as an isolated thrombocytopenia resulting from immune causes such as in infants born to mothers with immune thrombocytopenic purpura and in those with neonatal alloimmune thrombocytopenia.

In our study, the prevalence of pregnancy-associated thrombocytopenia was 5.2%, which is lower in comparison to other studies.^8,9,21^ Pregnancy-associated thrombocytopenia occurs in 6% – 10% of pregnant patients; however, its prevalence depends on its association with other medical conditions.^22^ The diagnosis of gestational thrombocytopenia is a diagnosis of exclusion and accounts for the majority of all cases of thrombocytopenia during pregnancy.^23,24^

### Limitations

Care should be taken in generalising the findings of the study because of the small numbers included in the study. The patient characteristics of those included may also differ from those seen at other levels of the healthcare system. Other limitations encountered were a lack of family history and other medications patients were taking, as this would have allowed for the classification and consideration of familial and drug-induced thrombocytopenia.

### Conclusion

In our study, thrombocytopenia was common in hospitalised patients compared to outpatients. Chemotherapy, sepsis and malignancies were the most common causes of thrombocytopenia. Focused investigations are warranted for prompt patient management. Knowledge of the prevalence and common aetiology of thrombocytopenia in a healthcare facility will assist clinicians in decision-making and interpretation of laboratory test results, leading to prompt and adequate patient management and potentially offsetting patient costs that may be incurred due to unwarranted laboratory investigations. Further research in this field is required at different levels of healthcare as differences in the reported prevalence of thrombocytopenia may be the result of differences in patient demographics and clinical presentation of patients, which may differ between these facilities.
